# Differential electron emission from polycyclic aromatic hydrocarbon molecules under fast ion impact

**DOI:** 10.1038/s41598-017-05149-8

**Published:** 2017-07-17

**Authors:** Shubhadeep Biswas, Christophe Champion, P. F. Weck, Lokesh C. Tribedi

**Affiliations:** 1 Tata Institute of Fundamental Research, Department of Nuclear and atomic Physics, Homi Bhabha Road, Colaba, Mumbai, 400 005 India; 2 Université Bordeaux 1, CNRS/IN2P3 Centre d’Études Nucléaires de Bordeaux Gradignan (CENBG) Chemin du Solarium, BP120, 33175 Gradignan, France; 3 Sandia National Laboratories, Albuquerque, New Mexico, 87185 USA

## Abstract

Interaction between polycyclic aromatic hydrocarbon (PAH) molecule and energetic ion is a subject of interest in different areas of modern physics. Here, we present measurements of energy and angular distributions of absolute double differential electron emission cross section for coronene (C_24_H_12_) and fluorene (C_13_H_10_) molecules under fast bare oxygen ion impact. For coronene, the angular distributions of the low energy electrons are quite different from that of simpler targets like Ne or CH_4_, which is not the case for fluorene. The behaviour of the higher electron energy distributions for both the targets are similar to that for simple targets. In case of coronene, a clear signature of plasmon resonance is observed in the analysis of forward-backward angular asymmetry of low energy electron emission. For fluorene, such signature is not identified probably due to lower oscillator strength of plasmon compared to the coronene. The theoretical calculation based on the first-order Born approximation with correct boundary conditions (CB1), in general, reproduced the experimental observations qualitatively, for both the molecules, except in the low energy region for coronene, which again indicates the role of collective excitation. Single differential and total cross sections are also deduced. An overall comparative study is presented.

## Introduction

Understanding of few-body collision dynamics has been a subject of intense research in past few decades. The first step towards this was the study of fast charge particle impact ionization of simple atoms and molecules. Nowadays, the extent is being extended to the studies of much more complex molecules of specific interests. For example, biologically relevant complex molecules impacted by energetic ions were investigated extensively from the perspective of hadron therapy^1–4^. The other problem, that has attracted a great deal of attention in recent times, is the understanding of several unidentified astrophysical features in the realm of microscopic details of the molecules which are present in the interstellar medium (ISM)^5^. In variety of widely contrasted interstellar (IS) environments a set of ubiquitous, unidentified infrared bands (UIR) are present in the emission spectra. Besides, the origin of features like diffuse interstellar bands (DIBs) in the absorption spectra in optical/near IR wavelength range, or the prominent 217.5 nm bump in the interstellar extinction curve are yet to be understood convincingly^5–8^. As far as present understanding, the evolution of different polycyclic aromatic hydrocarbon (PAH) molecules is highly plausible candidate to answer all the open questions regarding these unresolved features^5–10^.

PAHs are assembly of benzenoid rings of *sp* ^2^-hybridized C atoms. The peripheral atoms are attached to requisite number of H atoms. The remaining delocalized electrons together form a *π*-electron cloud over the C skeleton. This gives an extra stability to these molecules to their survival in the harsh IS environment. Generally, PAHs in ISM are formed in the outflows from C-rich giant stars. These can also be formed from the fragmentation of C dust particles in shocked regions and from photosputtering in diffuse IS clouds. Destruction of larger PAH complex in harsh IS environment is also an important production procedure for smaller PAHs. This continuous processing of PAHs is believed to be one of the important aspects of the evolution of the ISM, which is certainly related to the understanding of galaxy formation^9,10^. For example, the question of heating and cooling of the IS gases automatically came into picture since the first determination of temperature of the ISM by 21 cm emission/absorption line measurements. In this respect, C allotropes play an important role because these are the main suppliers of free electrons in the IS clouds, thus contributing to the heating of ISM^11^. The principal mechanism of the heating process is the removal of electron (ionization) from the allotropes by transferring energy from external energetic particles (cosmic rays) to the bound electrons. Then the suprathermal electrons produced in this way heat the other IS gases and eventually thermalize the whole system through elastic collisions. Here the radiative transition plays a minimal role^12^. Thus, in general, the study of electron emission mechanism of PAH molecules is an important issue which can shed light on our knowledge of different important astrophysical scenarios.

In the IS environment, cosmic ray (CR) is also an important component. It consists of protons, *α*-particles, heavier ions and electrons of energies spanning from few eV to hundreds of GeV. The sources of these energetic particles are different ISM shocks and supernova ruminants. The lower energy part of the spectrum, typically upto few hundreds of MeV, cannot be traced directly even with far ranging spacecraft, because these particles are excluded from the heliosphere or severely slowed down by the solar wind. But it is the most important part of the spectrum, as the intensity distribution sharply falls off with energy, and therefore the high energy (few GeV) cosmic rays have only marginal effect compared to those of few MeV^12^. Thus, it is extremely important to understand how the IS components are processed by these energetic ionic species, and this can only be possible through laboratory studies^5^. Particularly, it is instructive to study the interaction of MeV energy ions with PAH molecules as it can provide information about how the PAHs are processed by the low-energy cosmic ray in the ISM, and thus it helps to provide crucial inputs to various astrophysical models^13^.

Other than the astrophysical interest, the researchers are also quite fascinated about these molecules because of their collective electronic behaviour. It was predicted earlier that these molecules are capable of showing low energy (around 17 eV) plasmon like collective excitation following some external perturbation^14–16^. Nowadays, this idea has been tried to use in numerous important applications, like to fabricate novel plasmonic devices with tenability property^17^. Moreover, in some situations, it directly opens up new possibility of providing answers to some long standing problems in astrophysics like the origin of 217.5 nm extinction bump^18^. But fundamentally, unlike C_60_^19–23^ or metals^24^ or large nucleus^25^, PAHs are not well explored in the context of collective plasmon resonance. In this respect the study of electron emission from these molecules can enlighten the issue suitably, as electron emission channel is the fastest mode of de-excitation of these resonances^24^. Recently, the signature of such plasmon resonance ((*π* + *σ*) plasmon resonance around 17 eV) in coronene molecule has been shown in ion collision study^26^. Earlier, this was indicated in some of the photo-ionization studies^14,15^. But the question remains whether such plasmon excitation is also realizable in other PAH molecules or not.

**Figure 1:**
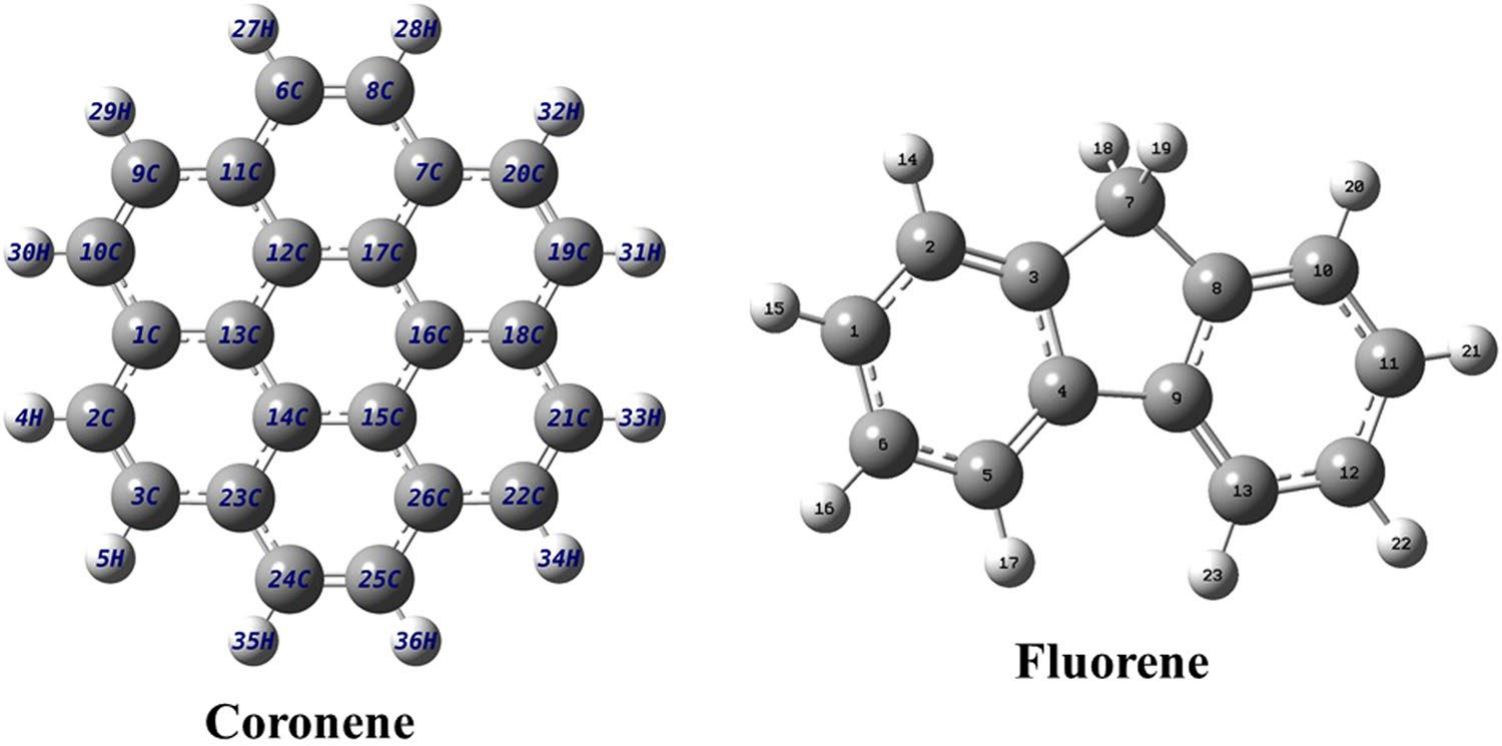
Structure of coronene and fluorene molecules. The dark shaded atoms at the centre part of the molecules are C atoms and the peripheral light shaded atoms are H atoms.

Keeping all these in mind, we have chosen two PAH molecules, namely coronene (C_24_H_12_) and fluorene (C_13_H_10_), which are different from each other with respect to their size as well as structure, to explore the aspects of ion-PAH collision. In particular, we intend to investigate the electron emission channels following energetic ion interaction. These are planner molecules of masses of 300 and 166, respectively. Coronene has seven six membered benzenoid rings, whereas fluorene consists of two six membered benzenoid rings connected by a five member ring (See Fig. 1). The numbers of valance electron in these are 108 and 62, respectively. For this study we carried out absolute double differential (in electron energy and emission angle) cross section (DDCS) $(\frac{{d}^{2}\sigma }{d\varepsilon d{\rm{\Omega }}})$ measurements of electron emission from these molecules under the impact of 3.75 MeV/u bare oxygen ions in a wide energy and angular ranges. It is worth mentioning that, to the best of our knowledge, this is the first detailed electron spectroscopy based measurement of PAHs perturbed by the energetic ions. Apart from photoionization and photofragmentation studies^27,28^, the existing literature of ion impact mainly includes post collision recoil-ion based measurements^29–36^. Other than that there are some reports available which demonstrate the energy loss mechanism of the energetic ionic projectiles within the PAHs^13,36–38^. In this article, in addition to the experimental results we also present calculations based on the quantum mechanical approach developed within the first-order Born approximation with correct boundary conditions (CB1). It is to be mentioned here that the calculations in quantum mechanical approach which requires a complete description of the molecular wavefunctions in term of the linear combinations of atomic orbitals (LCAO), give much more realistic description of the complex collision mechanism, but on the other hand, are very hard to perform. Such a comparison with *ab initio* model calculation is presented here for the first time to understand overall ion-PAH collision dynamics, it was not available in the earlier work on ion collision with coronene^26^, which mainly focused on the plasmon excitation aspects. It may also be mentioned that the electron DDCS measurements provide more stringent test to the theoretical models compared to single differential or total cross section measurements. Moreover, for comparison purpose, we also provide the results of similar measurements for smaller atomic and molecular systems with the same projectile specifications. This particularly helps us to discuss about the effects of the collective mode of the PAH molecules on the electron emission spectrum.

## Results

### Electron DDCS: Energy distribution

**Figure 2:**
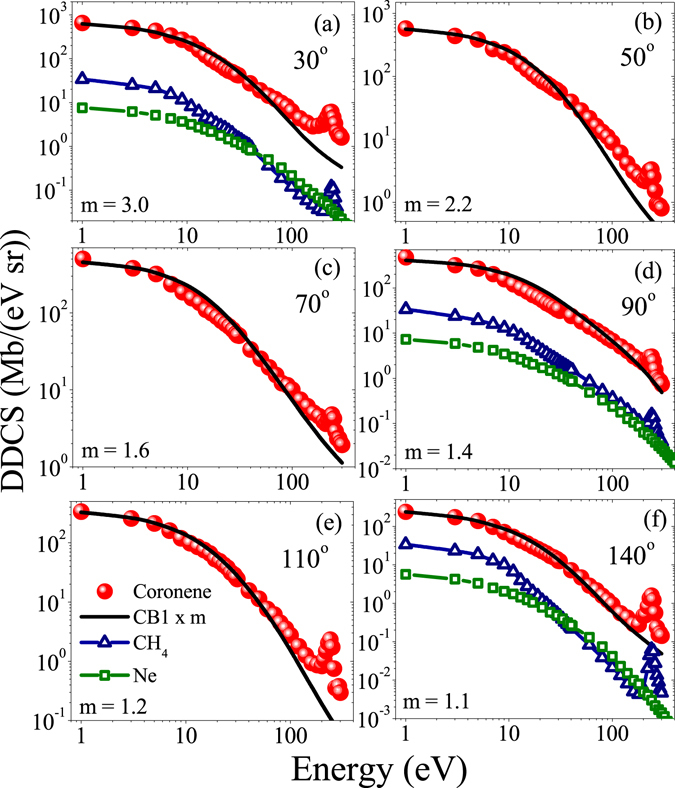
Energy distributions of absolute DDCS for coronene (circle), CH_4_ (triangle) and Ne (square) targets at different emission angles. Solid line represents the CB1 calculation (scaled by the factor m).

In Fig. 2, the energy distributions of the absolute DDCS at six representative emission angles are shown for coronene. The data for Ne and CH_4_ targets are also displayed at some of the plots along with the coronene data for comparison. The solid line in each plot represents the CB1 calculation for the coronene target. Apart from the fact that the cross sections for the Ne and the CH_4_ are always smaller than that of the coronene, in general, the qualitative behaviour of the distributions are observed to be very similar to each other, i.e. a rapid decrease in cross section with increasing electron energy. The low energy part of the spectrum, namely the soft collision region, is caused by the large impact parameter collisions involving low momentum transfer. In each of these plots, the broad peak around 240 eV corresponds to *K*-*LL* Auger electron emission from C atoms. The clearer visibility of the peak in the backward angles compared to the forward and the intermediate angles is due to the fact that the cross section corresponding to the Coulomb ionization continuum is less at backward angles compared to the other angle, whereas the *K*-*LL* Auger cross section distribution is almost isotropic in nature. This is very similar to the ion-atom collision cases^39^. Now as far as comparison with the CB1 calculation is concerned, it underestimates the experimental data for most of the electron energy regions at all angles. The factor (m) by which it underestimates decreases from about 3.0 to 1.1 with increasing value of emission angle. But, qualitatively it reproduces the features of the observed distributions quite well except only the *K*-*LL* Auger peak part which, being a characteristic relaxation process, is out of the scope of this model calculation. A further closer look into the plots reveals that the slopes of the theoretically predicted distributions, especially after around 20 eV, are little steeper than that of the observed distributions for all angles.

**Figure 3:**
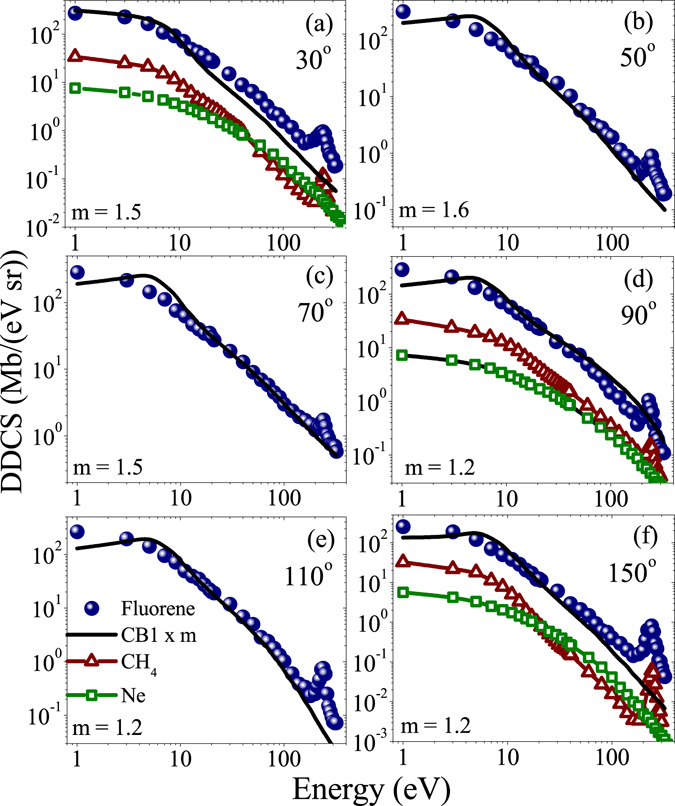
Energy distributions of absolute DDCS for fluorene (circle), CH_4_ (triangle) and Ne (square) targets at different emission angles. Solid line represents the CB1 calculation (scaled by the factor m).

In Fig. 3, similar DDCS energy distributions are shown for fluorene. In this case the qualitative features of the distributions are very similar to that of the above case. If we compare the absolute values of cross section between coronene and fluorene, it is always a factor of about 2.0 less for the later compared to the former. This can be qualitatively understood from the sizes of the molecules or numbers of valance electron. Regarding the comparison with the theory, the CB1 predicted curve is slightly different than the previous case in terms of overall characteristics. It shows a flat behaviour or slightly increasing trend at some angles upto around 8 eV. This does not match with the experimental observation of smoothly decreasing trend, and also this does not match with our expectation. After that it exhibits usual decreasing trend with the energy. In absolute scale, it underestimates the cross section by a factor (m) of 1.2 to 1.5, which was also the case for coronene.

**Figure 4:**
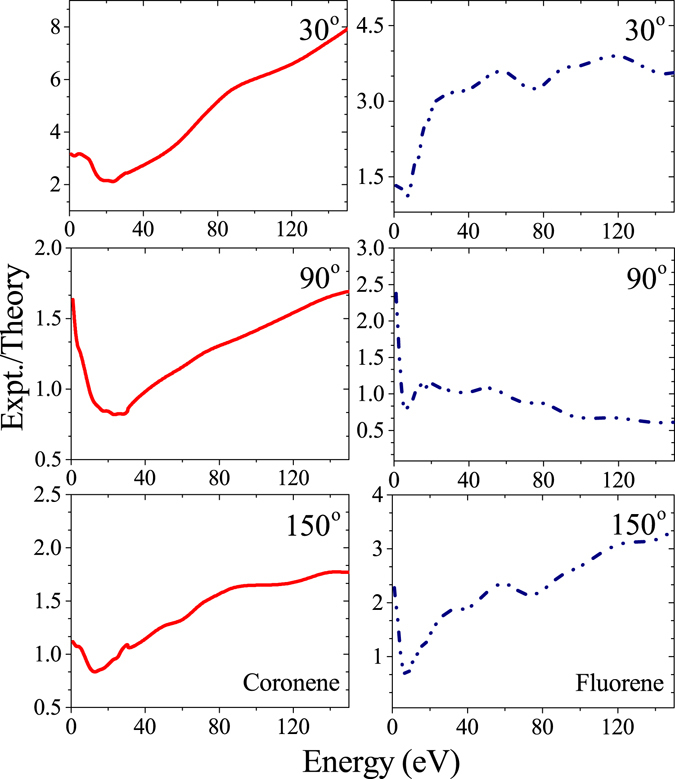
Energy distributions of ratio of DDCSs corresponding to experimental data and CB1 calculation. The left and right panels correspond to coronene and fluorene, respectively.

The overall comparison with the theory would be more clear from Fig. 4, where the ratio of experimental and theoretical DDCS values are plotted as a function of electron energy for both coronene and fluorene. For coronene, the deviation is minimum in the 20–30 eV region for all angles. At intermediate and backward angles, in this region the theory slightly overestimates the experimental data, whereas in the rest of the region it underestimates the data. After that specified region the deviation increases with electron energy. In general, at intermediate and backward angles the agreement between the theory and the experiment is better than that at the forward angle. In case of fluorene, the overall discrepancy is less compared to the case of coronene. Here the agreement is best at around 8 eV, and at the intermediate angle compared to the other angles. At extreme angles the deviation increases with electron energy.

### Electron DDCS: Angular distribution

**Figure 5:**
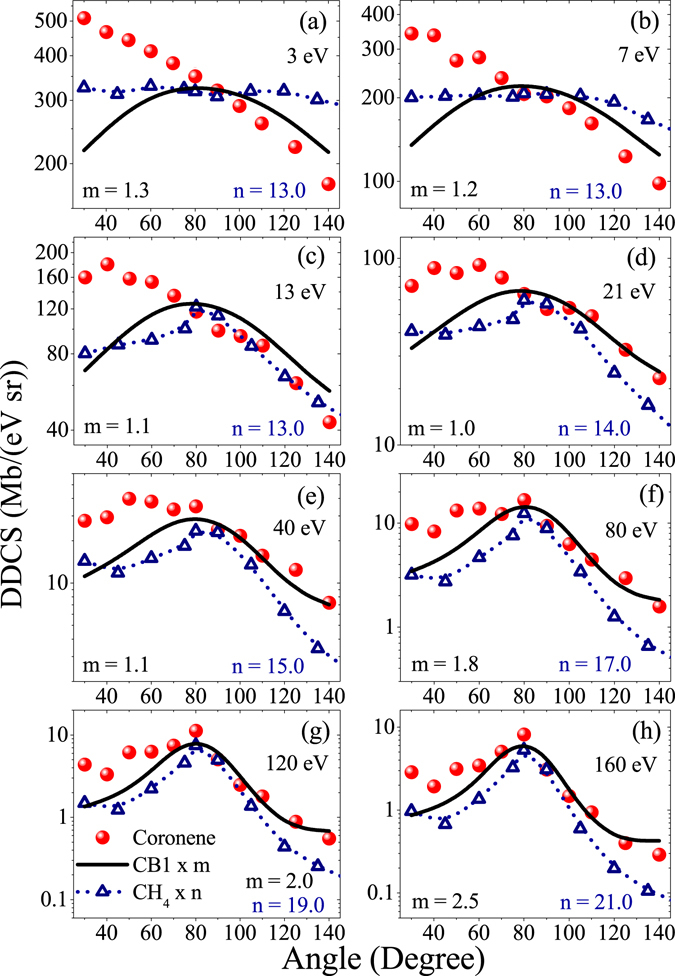
Angular distributions of absolute DDCS for coronene (circle) and CH_4_ (triangle) targets for different electron energies. Solid line represents the CB1 calculation. The CB1 curve and the data for CH_4_ are scaled by the factors m and n, respectively for representation.

The angular distributions of absolute DDCS for coronene are presented in Fig. 5 for eight representative electron energies. The distributions for CH_4_, scaled by a factor n, are plotted together for comparison. The CB1 calculation, which had to be also scaled by a factor m, is represented by the solid line. At lower energies upto around 20 eV, the distributions for coronene are steeply falling with increasing emission angle. This is in sharp contrast to the distribution observed for CH_4_. It is almost flat for 3 eV and 7 eV. After that a hump like structure at the intermediate angles due to binary nature collision arises. This behaviour is very similar to the ion-atom collision case^39,40^. The main striking difference between these two distributions is the associated forward-backward angular asymmetry, i.e. the relative difference between the cross sections corresponding to the forward and the backward angles. For example, at 7 eV, for coronene, the forward angle DDCS is about 3.2 times higher than the backward angle DDCS, whereas this value is only 1.2 for CH_4_. As electron energy increases, the amount of contrast comes down. At higher energies, the angular distributions for coronene are very similar to that of the CH_4_. In both the cases the peak at the intermediate angles corresponding to the binary encounter collision becomes prominent. The forward-backward angular asymmetries are also observed to be very similar for both the cases. It is quite large compared to that for lower energies. This large degree of angular asymmetry in the higher energy region is well understood in terms of projectile post collision effect^39,40^. As discussed earlier, the CB1 calculation underestimates the data almost for all energy ranges. In the low energy part of the spectrum, it also fails to reproduce the qualitative features of the observed angular distribution. It predicts an angular distribution which is expected from the binary nature of collision. The calculated forward-backward angular asymmetry is also very negligible compared to the observed one. As the value of the electron energy increases, the agreement becomes much better. After about 100 eV, it reproduces the qualitative features of the observed angular distributions well, especially at the intermediate angular region. This region is dominated by the binary encounter process, i.e. purely a two-body collision process. As a result it is well expected that the calculation based on quantum mechanical first-order approximation like CB1 would give better results. But at extreme angles the agreement is relatively worse. Mainly it fails to reproduce the observed forward-backward angular asymmetry. It predicts much smaller forward-backward angular asymmetry compared to the observed one.

**Figure 6:**
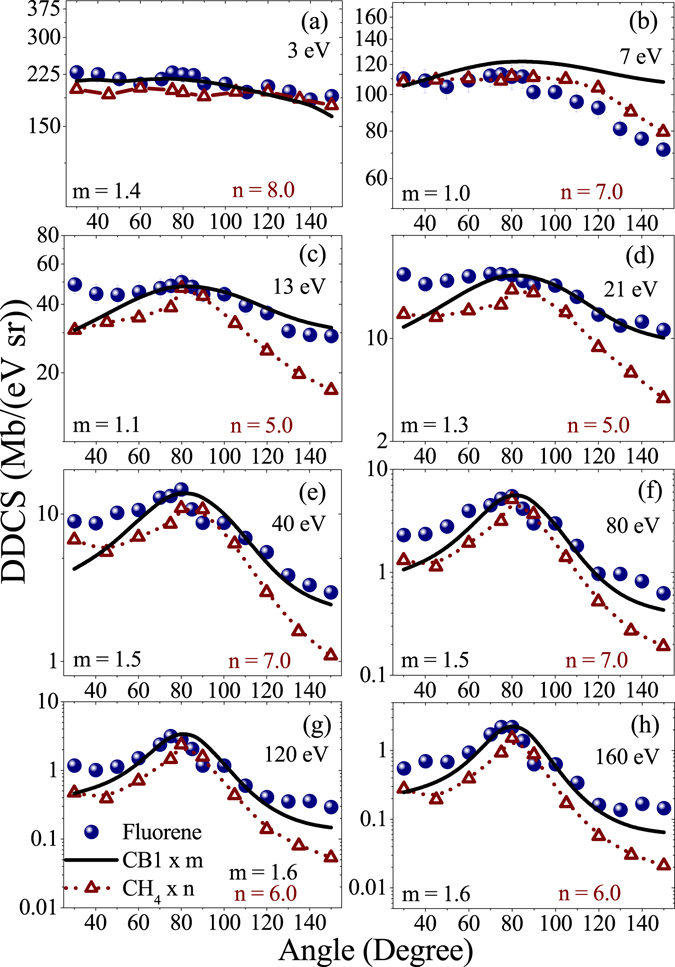
Angular distributions of absolute DDCS for fluorene (circle) and CH_4_ (triangle) targets for different electron energies. Solid line represents the CB1 calculation. The CB1 curve and the data for CH_4_ are scaled by the factors m and n, respectively for representation.

In Fig. 6, similar angular distributions of DDCS are shown for fluorene along with the distributions given by the theory and the CH_4_. At low energies, the nature of the distributions is different in several aspects from the case of coronene. In this case it is much more flatter distribution compared to coronene and very much similar to the case of CH_4_. Naturally, the associated forward-backward angular asymmetry is much lower. After 13 eV, it is even less than that for CH_4_ and it starts exhibiting the features of binary nature of collision, i.e. the hump like structure at the intermediate angles. Overall, qualitatively, the distributions for all energies are very similar to the cases of CH_4_. Though there are qualitative differences between low energy distributions of coronene and fluorene, the angular distributions corresponding to the higher energy electrons are quite similar to each other, mainly manifesting the features related to binary nature of collision and large forward-backward angular asymmetry. In this case the qualitative agreement with the distributions predicted by CB1 calculation is much better compared to the case of coronene. Here the low energy distributions are also quite well reproduced by the theory, relative to the earlier case. The main limitation is observed in the discussion of forward-backward angular asymmetry. Here also the calculation predicts much less forward-backward angular asymmetry compared to the observed one. Other than that, a good agreement is seen at most of the angular regions, especially at the intermediated angular region.

### Forward-backward angular asymmetry

**Figure 7:**
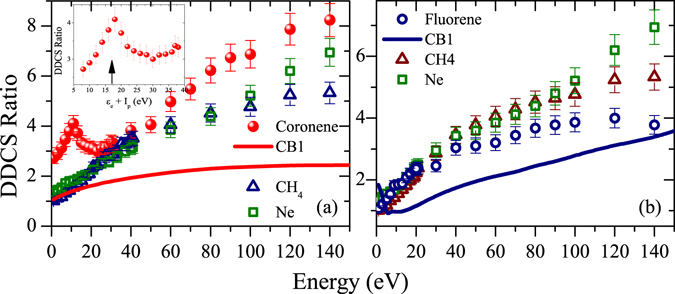
Energy distributions of DDCS ratio (forward to backward) for coronene (**a**) and fluorene (**b**). Data for other simpler targets are also shown. Solid lines represent the CB1 calculations. Inset in (**a**) shows the coronene plasmon peak at the plasmon excitation energy, i. e. *ε*_*e*_ + *I*_*p*_.

As seen in the above section, the forward-backward angular asymmetry is an important aspect of the electron angular distribution, which differs with target to target. Detailed analysis of it can give crucial information about the underlying mechanism of electron emission. To have a quantitative understanding of this, in Fig. 7(a,b), we have plotted the energy distributions of DDCS ratio signifying the enhancement of cross section at forward angle relative to backward angle, for coronene and fluorene, respectively. The angle 30° is chosen as forward angle and as backward angle 140° and 150° are chosen for coronene and fluorene targets, respectively. Similar distributions for other simpler targets, i.e. Ne and CH_4_, are also plotted in the same plots for comparison. If we first look at the distributions of Ne and CH_4_, these are qualitatively very similar to each other. In general, the ratio value increases monotonically from very low value to the higher value. The low value for the low energy electrons is due to nearly isotropic angular distribution, which is caused by the target centre effect. As these electrons are emitted in large impact parameter collisions, their distributions would be mainly governed by the effective Coulomb field of the residual target ion. On the other hand, the monotonically increasing behaviour is generally attributed to the projectile post collision effect. Because of the post collision projectile Coulomb field, the ionized electrons are dragged in the forward direction by the highly charged ionic projectile, which in turn increases the cross section in the forward direction compared to the backward direction. These higher energy electrons essentially move in a region of combined Coulomb fields of both the projectile ion and the residual target ion. This influence increases with increasing electron energy, because compared to the low energy electrons the velocities of the higher energy electrons are closer to the velocity of the projectile ion. As a result these are more influenced by the projectile Coulomb filed compared to the others^39,40^.

Now, if we look back to the distribution for coronene (Fig. 7(a)), we realize that the nature of the distribution is quite different than that of the above mentioned targets. In this case, it starts from a slightly higher ratio value and then forms a broad peak like structure at around 10 eV. Then 30 eV onwards it again follows an increasing trend and matches with the distributions corresponding to Ne and CH_4_ targets. Thus, though the projectile post collision effect picture, which generally works very well for the simpler targets, seems to be adequate to explain the behaviour in the higher energy region, the peak structure in the low energy region can not be explained by this and the target centre emission picture. It can be explained taking into account of collective plasmon excitation. For this molecule the predicted (*π* + *σ*) plasmon resonance energy is around 17 eV^16^. Therefore, we can see the signature of plasmon resonance in the emitted electron spectra at around $\hslash {\omega }_{plasmon}-{I}_{p}\sim 10\,{\rm{eV}}$, since the ionization potential (*I*_*p*_) is 7.29 eV (1^*st*^) for coronene (in gas phase). The observed peak position, here, very closely matches with this expected position of the plasmon electron signature^16,26^. In the inset of the figure it can be seen to match with the exact plasmon excitation energy, i. e. 17 eV. According to ref.^26^, this plasmon signature is visible in terms of angular asymmetry of electron distribution because of the oscillatory nature of plasmon resonance, which is perturbed by strong perturbing field of ionic projectile. This has been shown through a photo electron angular distribution model, which is calculated for large perturbation strength of the ionizing system^41–43^.

In case of fluorene, the qualitative features of the distribution are quite different from that of the coronene, especially in the low energy region, but very much similar to the simpler targets. It shows a monotonically increasing behaviour with increasing electron energy, though it takes the lowest value after about 40 eV among all the studied targets. Overall there is no noticeable feature in the distribution, which is quite different from the behaviour shown by the simpler targets or from our present understanding. More specifically the plasmon peak, similar to the case of coronene, in the low energy region is not visible here. Quantitatively, fluorene shows lesser asymmetry compared to that of coronene throughout the entire energy range.

Now, in these cases the distributions predicted by CB1 calculation are indicated by solid lines. For coronene, in general, it fails to reproduce the observed asymmetry. It predicts much lower value of the ratio than the observed one throughout the entire energy scale. It also does not predict any peak structure in the low energy region. The failure of reproducing the plasmon peak can be justified because this calculation does not incorporate any plasmon kind of collective excitation. For fluorene also, the CB1 calculation fails to reproduce the observed distribution well. It suggests lower value asymmetry than the observed one for most of the energy region. At extreme low energy region it shows a increasing trend with decreasing energy, which is not expected normally. In general, the underestimation of the asymmetry in both the cases, especially in the higher energy region, may be because of the fact that it is not a two centre distorted wave theory. In the distorted wave theories the effect of projectile Coulomb field before and after the collision is suitably incorporated, because of which the results are expected to be relatively better^39,40^. But this kind of calculation for these complex collision systems is hard to perform.

### Comparison between coronene and fluorene

**Figure 8:**
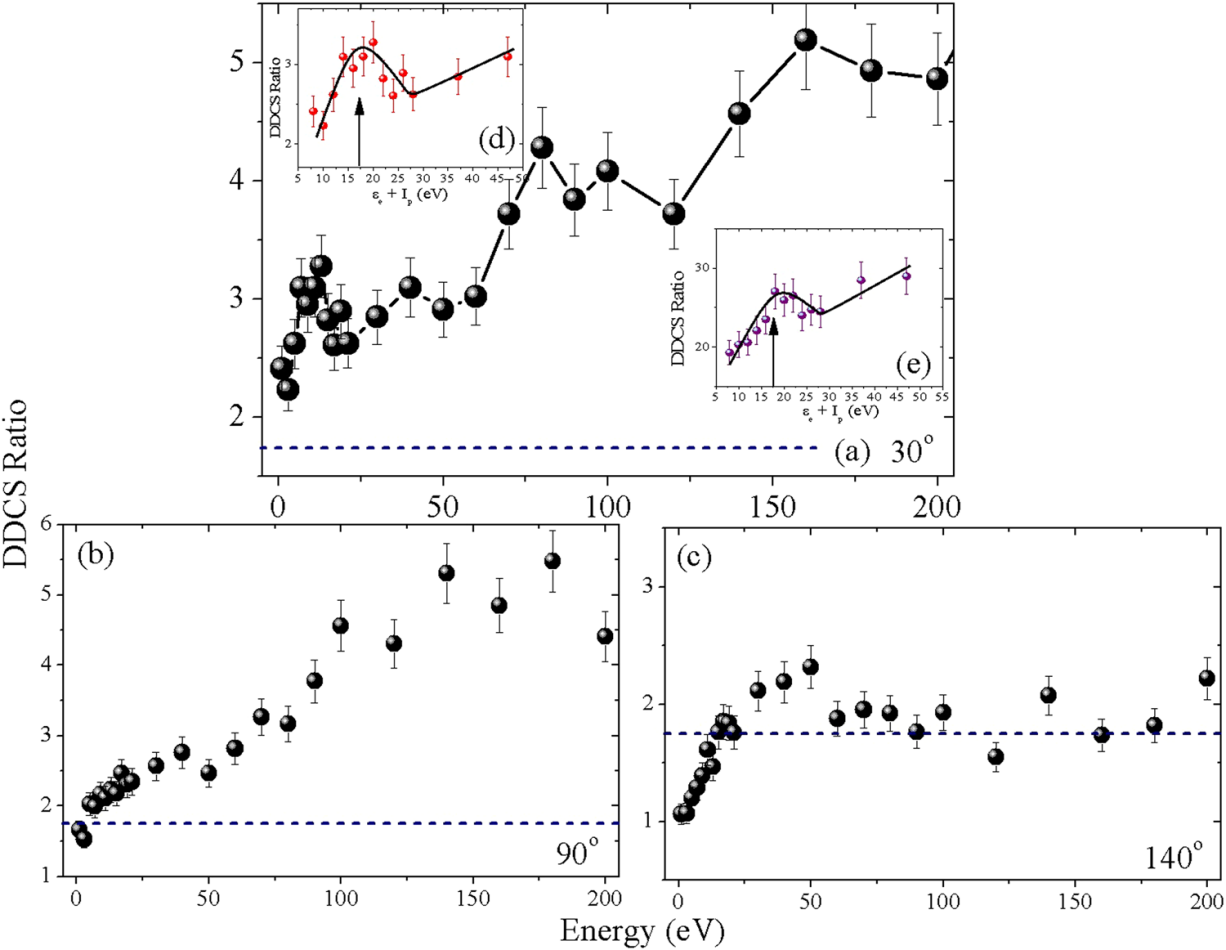
(**a–c**) Energy distributions of DDCS ratio (coronene DDCS to fluorene DDCS). The dashed horizontal line represents the value 1.74, which is the ratio of numbers of valance electrons in these two targets. Insets of (**a**) DDCS ratio of coronene and fluorene in the plasmon energy region where the energy scale is shifted by *I*_*p*_ (i.e. *ε*_*e*_ + *I*_*p*_). (**e**) DDCS ratio corresponding to coronene and CH_4_ in the plasmon energy region. The vertical arrows indicate the position of the plasmon excitation energy, i. e. 17 eV. Solid lines are drawn to guide the eye.

In Fig. 8(a–c), DDCS ratios corresponding to coronene and fluorene are shown for different angles. In forward and intermediate angles, in general, the ratio value increases with electron energy. This value reaches to a value as large as about 5.0 at round 200 eV. On the other hand at backward angle, it does not show increasing trend. After showing slightly increasing behaviour upto around 25 eV, it saturates at the value around 1.8, with slight fluctuation around it. It should be mentioned here that the number of valence electrons in coronene is 1.74 times more than that for fluorene. This is indicated by the dashed horizontal line in the plots. At the backward angle the DDCS ratio follows this line very well for most of the energy region. But the cross section ratios at forward and intermediate angles do not show similar scaling behaviour. The reason behind this is not fully understood. At the forward angle, in addition to the increasing trend, the peak structure corresponding to the plasmon excitation in coronene is prominently visible. This is clearly shown in the inset (d). The peak position very well matches with the expected plasmon energy i. e. 17 eV, as has been shown in the forward-backward ratio (in previous figure and ref.^26^). Additionally we also take the similar ratio with CH_4_ data, instead of fluorene. This is plotted in other inset (e), which also confirms the observation of plasmon peak at 17 eV. The contrbution of plasmon excitation is quite strong in case of forward electron emission and almost negligible for backward angle, as shown in ref.^26^. The observed nearly flat behaviour of the ratio around 1.74 [in Fig. 8(c)] for backward angle is consistent with this picture i.e. negiligible plasmon contribution. The sharp increase in the ratio values at higher electron energies in case of forward angles can not be explained at this stage.

### Single differential cross section (SDCS) and total cross section (TCS)

**Figure 9:**
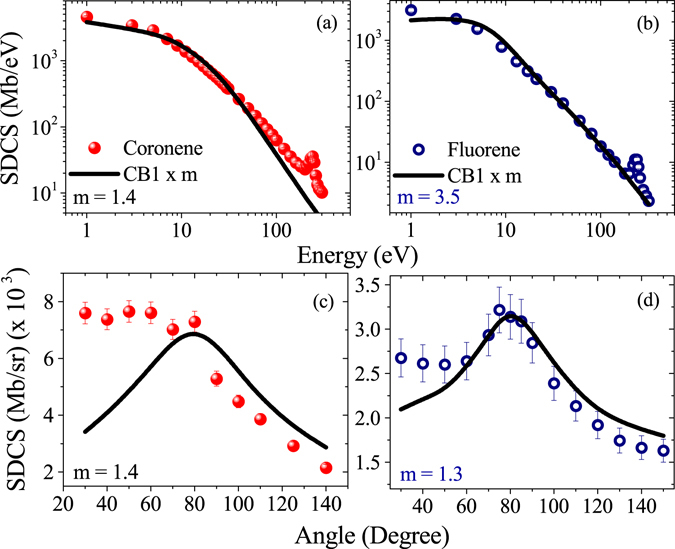
Energy and angular distributions of SDCS for coronene (left column) and fluorene (right column). Solid lines represent the CB1 calculations (scaled by the factor m).

To understand the overall angular and energy distributions of the emitted electrons, the SDCSs have been derived by integrating the DDCS spectra. In Fig. 9 its energy and angular distributions are displayed. For both the targets, the overall qualitative features of the energy distributions are very similar to that of the DDCS distributions, and these are very similar to each other also. Here the C K-LL Auger peaks are clearly visible. Though the gross trends of the distributions for both the targets are well reproduced by the CB1 calculation except the Auger part, quantitatively it underestimates the experimental data through out the entire energy region.

In contrast to the energy distribution, the angular distributions of SDCS corresponding to these targets are quite different from each other. For coronene, the forward angle cross sections are seen to be much higher compared to the backward angle cross sections. The usual feature of binary encounter peak is almost absent. It suggest that the low energy features in the DDCS level mainly govern the overall angular distribution. On the other hand, for fluorene, it is like ion-atom collision behaviour. There is forward-backward angular asymmetry present, but the binary encounter peak is prominently visible. The amount of forward-backward angular asymmetry is also much less compared to that of coronene. At the extreme backward angles, the cross sections for these targets are almost same with each other.

The prediction of the CB1 calculation, for coronene, does not match with the observation. Apart from the fact that it underestimates the cross sections as mentioned earlier, it provides a distribution which is very similar to the cases of the atomic targets^39,40^. It presents a prominent binary encounter peak at the intermediate angular region and the forward-backward angular asymmetry is much smaller than the observed one. For fluorene, besides the fact that it underestimates the experimental data, qualitatively it reproduces the angular distribution relatively well. There are some discrepancies mainly for the estimation of forward-backward angular asymmetry. It predicts more symmetric distribution than the observed one.

The TCS obtained from the experimental data for coronene and fluorene are about 56.729 Gb and 26.642 Gb, respectively, whereas these values estimated by the CB1 calculation are about 38.243 Gb and 20.703 Gb, respectively. Evidently, the theoretical calculation underestimates the experimental TCS for coronene by factor a of about 1.5 and it is about 1.3 for fluorene.

## Discussion

To summarize, we have measured the energy and the angular distributions of absolute DDCS of electron emission from coronene and fluorene molecules, which are two of the important members of the PAH family, under the collision of 3.75 MeV/u O^8+^ projectiles. The energy distributions of DDCS for both the targets are seen to be similar and these match with the general trend of the ion-atom or simpler molecule collision. On the other hand, there are some differences in the angular distributions corresponding to these PAH molecules, particularly in the low energy region. The distributions for fluorene shows isotropic behaviour at low energies. At higher energies it shows the expected behaviour, which can be explained by the binary encounter collision picture and two centre effect. These behaviours are nearly similar to that of the simpler targets. In contrary, the low energy electron distribution for coronene shows an unusually large forward-backward angular asymmetry which is due to the plasmon excitation, as shown also earlier. On the other hand, in the higher energy region, the characteristics of electron emission distributions are similar to the cases of ion-atom or small molecule collision. The signature of plasmon resonance for the case of coronene is clearly visible as a peak structure in the energy distribution of forward-backward DDCS ratio, whereas, for fluorene, the DDCS ratio distribution does not show such prominent peak in the plasmon energy region. The non-visibility of such peak in case of the fluorene molecule can be due to the low oscillator strength of plasmon resonance. In comparison with the coronene, it has much less number of available electron to form a polarized electron cloud which can oscillate against the ionic cage and produces plasmon resonance. Therefore, the strength of the plasmon resonance in PAH molecules certainly scales with the size of the molecule or number of available electron that can participate in plasmon oscillation. The case of such resonance in C_60_^23^ also fits with this argument. We have also studied the ratio of DDCS of the two molecules which also indicates the presence of a peak exactly at the plasmon excitation energy. This is consistent with our earlier observation of the plasmon contribution as derived from the forward-backward DDCS ratio. This conclusion is also strenghened from the observed peak in the ratio obtained using coronene and CH_4_ targets.

In order to understandstand the general behaviour of the energy and angular distributions of the DDCS data we have compared these with quantum mechanical calculation developed within the first-order Born approximation with correct boundary conditions (CB1). However this model does not take into account the many-body correlations or plasmon excitation. For both the targets, qualitatively, except in the KLL-Auger-energy region, the energy distributions of the DDCS are well reproduced by this. Quantitatively, it underestimates the experimental data by a factor of about 1.1 to 3.0 and 1.2 to 1.5 for coronene and fluorene, respectively. All the features of the angular distributions of DDCS for fluorene are also reproduced well by the calculations. But in case of coronene, though there is a good qualitative agreement with the higher energy data, it fails to reproduce the experimentally observed behaviour in the low energy region. Mainly it is unable to reproduce the observed large asymmetry in the angular distributions. There is a need to have a model which can include the collective behaviour in such large molecules and the projectile post collision effect to explain the experimental observations. These studies, particularly the collective excitation or many body effects may also be referred to the study of ion collisions with large molecules, such as, the DNA-bases, fullerenes etc, besides the other members of the PAH family. Apart from these the overall understanding of the energy and angular distributions of the absolute DDCS, SDCS and the derived TCS of the electron emission in ion-PAH collision, that come out from this study, would be beneficial in terms of crucial input to different astrophysical models.

## Methods

### Overview of the experimental procedure

In the present experiment, electron DDCS measurements for coronene and fluorene molecules were carried out with 3.75 MeV/u O^8+^ ions. The use of bare highly charged ions ensures to work in a strong perturbation regime as well as get rid of other complicated interactions^44,45^. The effusive vapour jets of coronene and fluorene molecules (99% pure, Sigma-Aldrich) were prepared by heating these at around 200 °C and 50 °C, respectively. For coronene experiment, a heater assembly, consisting of an electrically controlled oven with a nozzle of aspect ratio 10 on top, whose position can be controlled by an XYZ manipulator, was placed within a high vacuum scattering chamber, whose base pressure was maintained better than 2 × 10^−7^ Torr. To shield the electron detector from thermal radiation, a water cooling jacket was put around the oven. For fluorene experiment, the molecular holder was kept out side the chamber. A metal tube was used to connect the nozzle with the holder. In this case it was possible like that because of higher vapour pressure. These experiments demand uniform flow of target molecules throughout the experiment. To ensure this, initially the oven temperature was raised very slowly and after achieving desired vapour density, the temperature was kept fixed. The *in situ* characterization of the target flow was done using a quartz crystal thickness monitor mounted on the top of the interaction region. It shows the thickness of the deposited layer of target molecule with time. From that the variation of deposition rate was calculate and it was observed to vary by about 8% over a period of about 16 hours, for both the molecules. The projectile ion beam, extracted from the BARC-TIFR 14 MV tandem Pelletron accelerator facility in Mumbai, India, was made incident on the target effusive jet. Special cares were taken to get rid of stray electric field and earth’s magnetic field which affect the low energy electron detection. The number of projectile ions was determined by detecting them by a Faraday cup. The emitted electrons were energy analysed by a hemispherical electrostatic analyser whose energy resolution is around 6%^46^, and these were detected by a channel electron multiplier. By rotating the spectrometer the angular distribution was determined. In the present experiments the electrons were detected in the energy range of 1 to 320 eV, and the spectra were taken at different angles between 30° and 150°. To get the information about the background, same measurements were done without effusive jet. From the measured electron count and by background subtraction the DDCSs were obtained from the first principle^46^.

To obtain the absolute value of the cross section, the exact determination of the number of target molecules at the interaction region is necessary. But in this kind of effusive jet experiment, it is difficult to get this number reliably. Without this the relative DDCSs can be determined from the electron counts and employing solid angle path length correction for jet geometry properly. To avoid this problem as well to raise the relative DDCS values to the absolute scale, we used a novel method. In another flooded chamber experiment, with the same projectile, we determined absolute total C *K*-*LL* Auger cross section (*σ*_*Abs*_(*C* − *KLL*, *CH*_4_)) for methane target by integrating the DDCS data twice in the Auger region. Similarly, the relative Auger cross sections (*σ*_*Rel*_(*C* − *KLL*, *PAH*)) for PAH targets were also determined from the relative DDCSs. In all the cases exponential baseline subtraction was done in the single differential cross section (SDCS) (d*σ*/d*ε*) level after performing proper fitting.

Now, since Auger electron emission is an inner-shell ionization process, we assumed that the C *K*-*LL* Auger emission cross section is the same for CH_4_ and PAH targets, scaled by the number of C atoms. From that equality condition we got the unknown normalization factor as,

1 $$N=\frac{{\sigma }_{Abs}(C-KLL,C{H}_{4})\times {n}_{C}}{{\sigma }_{Rel}(C-KLL,PAH)}$$

where *n*_*C*_ is the number of C atoms of the PAH molecules. As the continuum part of the DDCS spectra as well as the Auger peak were produced from same target density, jet profile, beam overlap and other unknown experimental parameters, we could use the same absolute normalization factor as well to raise the continuum part to the absolute scale.

2 $$Absolute\,DDCS\,for\,PAH=N\times Relative\,DDCS\,for\,PAH$$

Further details of the normalization procedure is available in ref.^2^. The overall uncertainty in these absolute cross section measurements were estimated to be about 10% to 15% which mainly arises from the target density fluctuation, normalization procedure and the statistical uncertainty.

### Theoretical Model: CB1

The cross sections presented in this work have been calculated within the first-order Born approximation with initial and final wave functions verifying correct boundary conditions. This approach, successfully used earlier by Champion *et al*.^47^ for investigating ionization of water^48–50^, can be interpreted as an extension of the CB1 model introduced by Belkić *et al*.^51^ for studying the electron capture from atomic targets.

In the laboratory frame, the triply differential cross sections (TDCS), namely, differential in the scattering direction (Ω_*s*_), differential in the ejection direction (Ω_*e*_) and differential in the ejected energy (*E*_*e*_), can be simply written as

3 $${\sigma }^{(3)}({{\rm{\Omega }}}_{s},{{\rm{\Omega }}}_{e},{E}_{e})\equiv \frac{{d}^{3}\sigma }{d{{\rm{\Omega }}}_{s}d{{\rm{\Omega }}}_{e}d{E}_{e}}=\sum _{j=1}^{N}\,\frac{{d}^{3}{\sigma }_{j}}{d{{\rm{\Omega }}}_{s}d{{\rm{\Omega }}}_{e}d{E}_{e}}\equiv \sum _{j=1}^{N}\,{\sigma }_{j}^{\mathrm{(3)}}({{\rm{\Omega }}}_{s},{{\rm{\Omega }}}_{e},{E}_{e}),$$

where *N* refers to the number of molecular orbitals used in the target description (*N* = 44 and 78 for fluorene and coronene, respectively). The current target description is based on the restricted Harthree-Fock method with geometry optimization (RHF/3-21G)^52^ and models each molecular subshell of the targets by means of linear combinations of atomic orbitals provided within a complete neglected differential overlap (CNDO) approach.

In these conditions, the TDCS for each molecular orbital labeled *j* can be expressed as a weighted sum of atomic triply differential cross sections ${\sigma }_{at,i}^{\mathrm{(3)}}$ corresponding to the different atomic components involved in the molecular subshell description, namely,

4 $${\sigma }_{j}^{\mathrm{(3)}}({{\rm{\Omega }}}_{s},{{\rm{\Omega }}}_{e},{E}_{e})=\sum _{i}\,{\xi }_{j,i}\cdot {\sigma }_{at,i}^{\mathrm{(3)}}({{\rm{\Omega }}}_{s},{{\rm{\Omega }}}_{e},{E}_{e}),$$

where *ξ*_*j*,*i*_ stand for the *effective* number of electrons of the different atomic orbitals and where the atomic triply differential cross ${\sigma }_{at,i}^{\mathrm{(3)}}$ are calculated by using the well-known expression, namely,

5 $${\sigma }_{at,i}^{\mathrm{(3)}}({{\rm{\Omega }}}_{s},{{\rm{\Omega }}}_{e},{E}_{e})={\mathrm{(2}\pi )}^{4}\cdot {\mu }^{2}\cdot \frac{{k}_{s}{k}_{e}}{{k}_{i}}\cdot {|{[{T}_{a,b}]}_{i}|}^{2}$$

In Eq. 5, [*T*_*a*,*b*_]_*i*_ denotes the atomic transition matrix element between an initial state *a* and a final state *b* with *μ* = *A*_*ion*_ · *M*_*p*_ where *A*_*ion*_ designates the projectile mass number and *M*_*p*_ the proton mass whereas **k**_*i*_, **k**_*s*_, and **k**_*e*_ represent the wave vectors of the incident ion, the scattered ion and the ejected electron, respectively.

Furthermore let us note that the collisional process is here described within the independent electron model (IEM) and employing frozen core approximation. These approximations have been successfully used for numerous ionization reactions (see for example refs^53,54^). It was shown that, the interaction between the projectile and the residual target plays a negligible role when doubly differential cross sections (DDCS) are determined as a function of the linear momentum of the emitted electron^55–57^. Now the DDCSs can be easily obtained after integration of the TDCS over the projectile scattering direction Ω_*s*_ with a transition matrix element ${[{\tilde{T}}_{a,b}]}_{i}$, namely,

6 $${[{\tilde{T}}_{a,b}]}_{i}=\langle {\phi }_{i,b}(R)\cdot {\varphi }_{i,b}(r)|\frac{{Z}_{P}}{R}-\frac{{Z}_{P}}{|R-r|}|{\phi }_{i,a}(R)\cdot {\varphi }_{i,a}(r)\rangle ,$$

where **R** and **r** give the positions of the projectile and of the active electron, respectively and *ϕ*_*i*,*a*_(*R*) and *ϕ*_*i*,*b*_(*R*) refer to incoming and outgoing projectile plane waves, whereas *φ*_*i*,*b*_(*r*) stands for the ejected-electron Coulomb wave function, respectively. *φ*_*i*,*a*_(*r*) represents the atomic wave function of the *i* ^*th*^-orbital used in the CNDO expansion of each target molecular subshell. These atomic wave functions refer to the C(*2p*) and H(*1* *s*) ones and are expanded on spherical harmonic basis with a radial part given in terms of Slater functions, namely,

7 $${\mathrm{\varphi ;}}_{i,a}(r)=\sum _{k=1}^{{N}_{i}}\,\frac{{\mathrm{(2}{\varsigma }_{k})}^{{n}_{ik}+\mathrm{1/2}}}{\sqrt{\mathrm{(2}{n}_{ik})!}}\cdot {r}^{{n}_{ik}-1}\cdot {e}^{-{\varsigma }_{ik}\cdot r}\cdot {Y}_{{l}_{ik}{m}_{ik}}({{\rm{\Omega }}}_{r})\equiv \sum _{k=1}^{{N}_{i}}\,{f}_{ik}(r)\cdot {Y}_{{l}_{ik}{m}_{ik}}({{\rm{\Omega }}}_{r}),$$

where *N*_*i*_ denotes the number of partial waves (*n*_*ik*_, *l*_*ik*_, *m*_*ik*_) used for the description of the *i* ^*th*^ atomic orbital. For more details about these coefficients, we refer the reader to the Clementi’s tables of atomic functions^58^.

Finally, it should be here mentioned that the $\frac{{Z}_{p}}{R}$ term in the perturbative potential of Eq. 6 corrects the Coulomb long range behavior of the interaction between the projectile and the active electron at their asymptotic separations.

Thus, by using the well-known partial-wave expansion of the plane wave as well as that of the Coulomb wave, we easily access to an analytical form of the TDCS which may be, as previously mentioned, converted into DDCS by means of a numerical integration over the projectile scattering direction, namely,

8 $$\begin{array}{rcl}{\sigma }_{at,i}^{\mathrm{(2)}}({{\rm{\Omega }}}_{e},{E}_{e}) & \equiv  & \int d{{\rm{\Omega }}}_{s}\cdot {\sigma }_{at,i}^{\mathrm{(3)}}({{\rm{\Omega }}}_{s},{{\rm{\Omega }}}_{e},{E}_{e})\\  & = & \frac{32{k}_{s}}{{k}_{i}{k}_{e}{q}^{4}}\int d{{\rm{\Omega }}}_{s}\cdot \sum _{k=1}^{{N}_{i}}\,[{(\frac{{X}_{ik}^{^{\prime} }}{4\pi })}^{2}+\sum _{\mu =-{l}_{ik}}^{{l}_{ik}}\,[|{Z}_{ik}{|}^{2}\\  &  & -\,\Re e(\frac{{Z}_{ik}}{\sqrt{\pi {\hat{l}}_{ik}}}{Y}_{{l}_{ik}\mu }^{\ast }\,({{\rm{\Omega }}}_{e})\,{i}^{{l}_{ik}}{e}^{-i{\sigma }_{{l}_{ik}}}{X}_{ik}^{^{\prime} })]]\end{array}$$

where *σ*_*l*_ denotes the Coulomb phase shift and $\Re e(z)$ the real part of the complex *z*, whereas

9 $$\begin{array}{rcl}{X}_{ik}^{^{\prime} }\equiv {X}_{ik}^{^{\prime} }({k}_{e}) & = & {\int }_{0}^{\infty }dr\cdot r\cdot {F}_{{l}_{ik}}({k}_{e},r)\cdot {f}_{ik}(r)\\ {Z}_{ik}\equiv {Z}_{ik}({k}_{e},q) & = & \sum _{l=0}^{\infty }\,\sum _{{l}_{1}=|l-{l}_{ik}|}^{l+{l}_{ik}}\,\sum _{{m}_{1}=-{l}_{1}}^{{l}_{1}}\,{i}^{l-{l}_{1}}\cdot {e}^{i{\sigma }_{{l}_{1}}}\cdot {X}_{ik}^{l{l}_{1}}\cdot {Y}_{{l}_{1}{m}_{1}}({{\rm{\Omega }}}_{e})\\  &  & \cdot \,{Y}_{l{m}_{1}-\mu }^{\ast }({{\rm{\Omega }}}_{q})\cdot {(-\mathrm{1)}}^{{m}_{1}}\cdot \sqrt{{\hat{l}}_{1}\hat{l}}\cdot (\begin{array}{l}{l}_{1}\,l\,{l}_{ik}\\ 0\,0\,0\end{array})\cdot (\begin{array}{l}{l}_{1}\,\quad \,\,\,\,l\,\quad {l}_{ik}\\ -{m}_{1}\,{m}_{1}-\mu \,\,\mu \end{array}).\end{array}$$

with $\hat{l}=2l+1$ and where *q* = *k*_*i*_ − *k*_*s*_ denotes the momentum transfer.

Note that in Eq. 9, *F*_*l*_(*k*_*e*_, *r*) refers to the radial hypergeometric function whereas ${X}_{ik}^{l{l}_{1}}({k}_{1},q)$ is expressed as

10 $${X}_{ik}^{l{l}_{1}}({k}_{1},q)={\int }_{\,0}^{\,\infty }\,dr\cdot r\cdot {F}_{{l}_{1}}({k}_{1},r)\cdot {j}_{l}(qr)\cdot {f}_{ik}(r),$$

where *j*_*l*_(*qr*) refers to the Bessel function.

Finally, note that in the present quantum mechanical calculations, the effective target charge seen by the escaping ejected electron is not equal to the asymptotic charge, namely, ${Z}_{T}^{\ast }=1$ but taken as ${Z}_{T}^{\ast }=\sqrt{-2{n}_{\alpha }^{2}{\varepsilon }_{\alpha }}$ where *n*_*α*_ refers to the principal quantum number of each atomic orbital component used in each MO expansion whereas the active electron orbital energy *ε*_*α*_ is related to the ionization energies *B*_*j*_(>0) of the occupied molecular orbitals by *ε*_*α*_ = −*B*_*j*_.
