# Author Correction: Differential electron emission from polycyclic aromatic hydrocarbon molecules under fast ion impact

**DOI:** 10.1038/s41598-017-17041-6

**Published:** 2017-12-04

**Authors:** Shubhadeep Biswas, Christophe Champion, P. F. Weck, Lokesh C. Tribedi

**Affiliations:** 1 Tata Institute of Fundamental Research, Department of Nuclear and atomic Physics, Homi Bhabha Road, Colaba, Mumbai, 400 005 India; 2 Université Bordeaux 1, CNRS/IN2P3 Centre d’Études Nucléaires de Bordeaux Gradignan (CENBG) Chemin du Solarium, BP120, 33175 Gradignan, France; 3 Sandia National Laboratories, Albuquerque, New Mexico, 87185 USA

*Scientific Reports* **7**:5560; doi:10.1038/s41598-017-05149-8; Article published online 17 July 2017

P. F. Weck was omitted from the author list in the original version of this Article. This has been corrected in the PDF and HTML versions of the Article.

The Acknowledgements section now reads:

The authors would like to thank the staffs at the BARC-TIFR Pelletron accelerator facility for smooth operation of the machine. We thank D. Misra for his various helps during the measurements. P.F.W would like to thank the Sandia National Laboratories which is a multi-mission laboratory managed and operated by National Technology and Engineering Solutions of Sandia, LLC., a wholly owned subsidiary of Honeywell International, Inc., for the U.S. Department of Energy’s National Nuclear Security Administration under Contract DE-NA0003525.

The Author Contributions section now reads:

L.C.T. had the idea and planned this experiment. L.C.T. and S.B. executed the experiment, analysis and interpretation. C.C. and P.F.W. provided the model calculations in which P.F.W. provided the wave functions of the molecules whereas C.C. contributed towards the CB1 model calculations. L.C.T.,S.B. and C.C. prepared the paper which was reviewed by all the authors.

